# Bilateral Second Arch Branchial Fistula-A Case Report 

**DOI:** 10.22038/ijorl.2019.35910.2186

**Published:** 2019-11

**Authors:** Liang-Chye Goh, Roslim-Siti Norain, Zulkifli Shifa, Anura-Michelle Manuel

**Affiliations:** 1 Department of Otorhinolaryngology, University of Malaya Medical Center, Kuala Lumpur, Malaysia.; 2 Department of Otorhinolaryngology, Hospial Sultanah Bahiyah, Alor Setar, Kedah, Malaysia.

**Keywords:** Branchial, Bilateral, Congenital, Excision, Fistula

## Abstract

**Introduction:**

Branchial arch anomalies represent defects in embryological developments whereby parts of the branchial arch persist in the head and neck regions as sinuses, fistulas, or cysts. These anomalies usually present as a unilateral lesion in the head and neck of young adults and children, which are excised upon the emergence of complications.

**Case Report:**

Herein, we presented a rare case of a 4-year-old child, who had been diagnosed with a complete bilateral second arch branchial fistula. The excision was made using the bilateral stepladder approach and tonsillectomy.

**Conclusion:**

The bilateral stepladder approach was a feasible method in excising a complete bilateral branchial fistula. However, larger-scale studies should be conducted on the surgical techniques of bilateral branchial fistulae excision in order to optimize the cosmetic outcome of the surgery.

## Introduction

The branchial arches are a series of six mesodermal pouches that develop at the side of the primitive pharynx within 4-7 weeks of gestation (1). The aforementioned arches serve as precursors to the development of the structures found in the face, neck, and pharynx. As the branchial arch develops, the failed obliteration of the branchial clefts often leads to the development of branchial arch anomalies, whereby they may present as cysts, sinuses, or fistulas (1). 

About 80% of cases present as branchial cysts, and the remaining 20% emerge as sinuses, fistulas, or cartilage remnants (2). Branchial arch anomalies are the most common congenital anomaly, affecting the neck, whereby the 2^nd^ branchial arch is the most frequently involved (1,2).

Branchial fistulas, wherein an epithelial tract connects the branchial pouch and cleft, are the uncommon manifestations of branchial anomalies. Such connections are often described as true or complete brachial fistulas. However, this is rarely the case, as most branchial fistulas present in incomplete forms (3). A complete 2^nd^ branchial arch fistula should have an internal opening at the tonsillar fossa and an external opening overlying the medial aspect of the sternocleidomastoid.

## Case Report

A 4-year-old boy with an unremarkable perinatal and medical history referred to us with the complaint of mucoid discharge from both sides of the neck over a duration of 6 months which had worsened upon the consumption of fluids. He had a few episodes of acute infections of the fistula which resolved upon the completion of a course of antibiotics. The patient had no family history of a similar diagnosis. 

**Fig 1:**
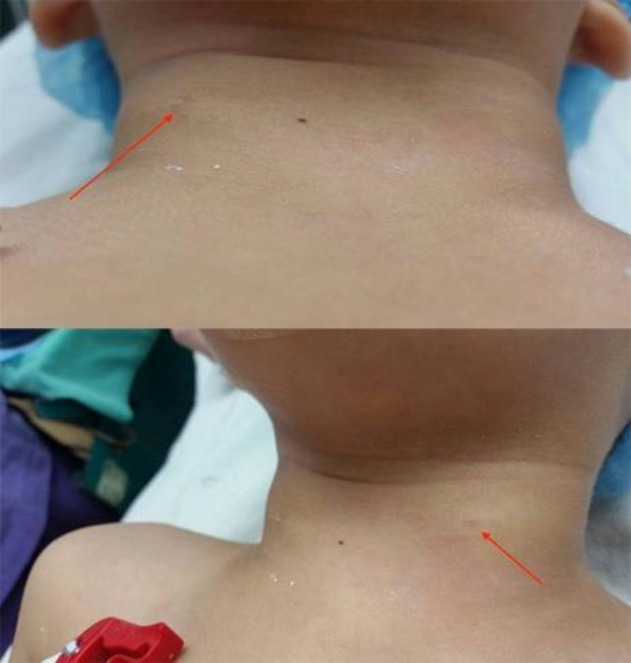
Preoperative photograph of the child showing the external openings of both branchial fistulas (Arrow)

On examination, he was healthy with no craniofacial deformities. A small pin-hole-sized defect was seen at about the junction of the upper 2/3 and lower 1/3 of the anterior border of the sternocleidomastoid muscle on both sides (Fig.1). There was a scanty mucoid-like discharge from both cervical fistulous openings. Oropharyngeal examination revealed bilaterally enlarged tonsils (grade 2 Friedman classification). In addition, the fibreoptic nasopharyngoscopic examination was unremarkable. Normal renal ultrasound findings and profile ruled out hypertrophic or atrophic kidneys in view of the possibility of kidney maldevelopment in branchio-oto-renal syndrome. 

**Fig 2:**
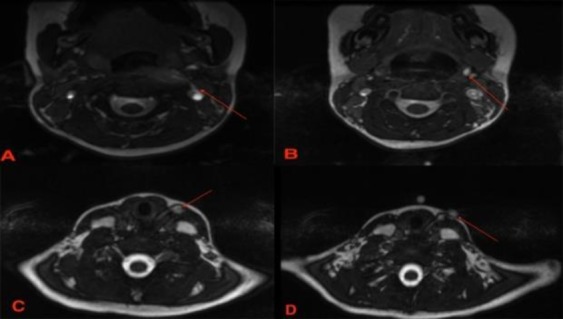
Intra-operative images of both branchial fistulas being removed using a stepladder incision

The external ear was grossly normal, and audiometric testing (e.g., tympanogram and pure tone audiometry) ruled out the presence of conductive or sensorineural hearing loss which may present as a component of branchio-oto-renal syndrome. The magnetic resonance imaging (MRI) of the neck with a fistulogram was carried out by injecting normal saline into the defect, while the images were read using T1 FIESTA sequence (Fig.2). The MRI images showed the evidence of a complete bilateral branchial fistula. An excision was then performed under general anesthesia after 2 weeks. 

**Fig 3:**
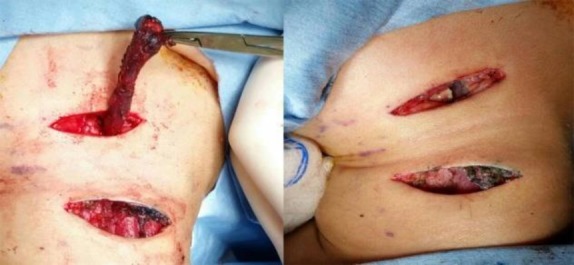
Removed specimen of both branchial fistulae with the arrows pointing at the internal opening of the branchial fistula; A) left branchial fistula and B) right branchial fistula

The child was placed supine with the neck extended, under general anesthesia. A transverse elliptical incision was made around the cervical fistulous opening, dissected deep to the subplatysmal plane. The dissection was continued superiorly, tunneling under the subplatysmal layer to follow the path of the fistulous tract. The second transverse incision was made just above the level of the hyoid in a stepladder fashion to facilitate further superior dissection (Fig.3). 

Identification of the tract was aided by instilling methylene blue into the tract using the cannula of a 26G branulla. The tract was located between the external and internal carotid arteries, superficial to the hypoglossal nerve and deep to the digastric muscle. The internal opening was identified at the posterior pillar of the ipsilateral tonsillar fossa superiorly. An elliptical incision was made around this opening, and the tract dissected inferiorly to meet the cervical dissection. 

**Fig 4:**
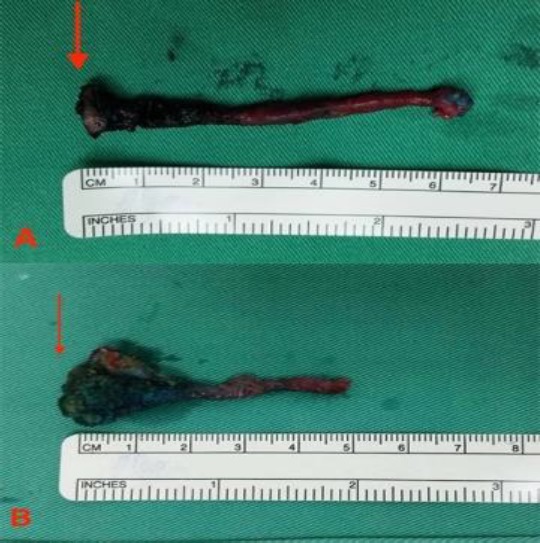
Magnetic resonance image of pre-operative fluid attenuated inversion recovery showing the axial views of saline being injected into the left branchial fistula; A-D) a complete fistula starting from the left tonsillar fossa to the skin overlying (red arrow) the left anterior neck

The whole tract was completely excised and delivered out through the upper cervical incision (Fig.4). The procedure was then repeated on the right side of the neck which had a fistulous tract of similar anatomical extent. The subcutaneous and skin layers were sutured using absorbable and non-absorbable suture materials (Polyglactin 4/0 and polyamide 4/0).

The patient was discharged with no post-operative complications after 3 days. Histopathological examination of the excised lesion revealed the features of respiratory epithelium and confirmed the absence of malignancy and tuberculous infection. On follow-up performed 12 months after the surgery, there was no evidence of recurrence.

## Discussion

Branchial arches are ectoderm-lined clefts which develop from the branchial apparatus as a part of a series of arches, pouches, and grooves which extend into the oral cavity. During embryonic development, as the second arch grows caudally, it fuses with the 3^rd^ and 4^th^ arches to form a deep groove before joining the skin as an external opening, thereby being termed cervical sinus. Persistence of this fistula is often due to the breakdown of endoderm during embryogenesis, and this forms a tract to the skin at the junction of lower 1/3^rd^ and upper 2/3^rd^ of the anterior border of the sternocleidomastoid of the affected neck (4).

The 2^nd^ branchial arch fistula is a rare manifestation of branchial arch anomalies as most cases are three times more likely to present as branchial cysts in young adults with an almost equal gender distribution (5,2). In a large series conducted by Chionh et al., about 15% of cases presented in children aged <10 years, while the majority of cases presented at the age range of 10-40 years (6). 

Most cases of complete 2^nd^ branchial fistula often present in childhood (2,4). These cases emerge as a persistent unilateral discharging defect usually at the right side of the neck which worsens upon the consumption of drinks (7). As bilateral branchial fistula and sinuses are rare, they are often associated with a family history in about 6% of cases (8-10). Nonetheless, about 2% of sporadic cases are associated with an incidence (11).

Branchial fistulas are often diagnosed intra-operatively as investigations like fistulograms often fail to reveal the full extent of the fistulous tract, as observed in our case. It is useful but sometimes difficult to differentiate between the 2^nd^ arch and 3^rd^/4^th^ branchial arch anomaly due to various reasons. Some of these reasons could be the presence of granulation tissue, secretions within the lumen of the fistula, or tight muscle surrounding the tract (5). 

Therefore, the extent of a fistulous tract is best made intra-operatively when muscle relaxants are given, and the tract can be traced using a dye. Special considerations should be also given to clinical differentials, such as malignancies and tuberculous fistulas, which often present in the neck. Complete surgical excision of the branchial fistula remains the cornerstone of treatment (12). 

Two conventional methods have been mentioned in the literature, namely the stepladder approach and single-incision approach (5,7). In the present study, a bilateral stepladder approach was employed in the management of this case for the purpose of better surgical access. This method was associated with good surgical outcome with no recurrence after 2 years of follow-up. 

The single-incision approach should be reserved for branchial sinuses or branchial fistulas with a short tract. The management of bilateral branchial fistulas follows the same principals of unilateral fistula or sinus tract excision wherein complete tract excision is mandatory to prevent recurrent symptoms.

## Conclusion

In conclusion, we present the case of a 4-year-old patient with bilateral complete branchial fistulas who had undergone a surgical excision using a bilateral stepladder incision with good clinical and post-operative outcome. The authors would like to advocate the need for further studies on the excision technique of a “complete” bilateral branchial fistulae as the post-operative cosmetic outcome could have possibly been improved upon.
